# Distinct Symptom Occurrence Subgroups Among Patients With Colorectal Cancer: Differences in Social Determinants of Health and Diet Quality

**DOI:** 10.1002/nur.70083

**Published:** 2026-05-26

**Authors:** Youran Lee, Qing Yang, Melissa Kay, Donald E. Bailey, Biljana Gigic, Sheetal Hardikar, Adetunji T. Toriola, David Shibata, Christopher I. Li, Doratha A. Byrd, Jane C. Figueiredo, Ildiko Strehli, Megan Mclaws, Vaia Florou, Mmadili Ilozumba, Patricia Erickson, Apurva Medhe, Cornelia M. Ulrich, Rosa M. Gonzalez‐Guarda

**Affiliations:** ^1^ School of Nursing Duke University Durham North Carolina USA; ^2^ School of Nursing University of Michigan Ann Arbor Michigan USA; ^3^ School of Medicine Wake Forest University Winston‐Salem North Carolina USA; ^4^ Department of General, Visceral, and Transplantation Surgery Heidelberg University Hospital Heidelberg Germany; ^5^ Huntsman Cancer Institute University of Utah Salt Lake City Utah USA; ^6^ Division of Public Health Sciences, Department of Surgery Washington University School of Medicine and Siteman Cancer Center St Louis Missouri USA; ^7^ Department of Surgery University of Tennessee Health Science Center Memphis Tennessee USA; ^8^ Division of Public Health Sciences Fred Hutchinson Cancer Research Center Seattle Washington USA; ^9^ H. Lee Moffitt Cancer Center and Research Institute Tampa Florida USA; ^10^ Samuel Oschin Comprehensive Cancer Institute Cedars‐Sinai Medical Center Los Angeles California USA; ^11^ Department of Population Health Sciences University of Utah Salt Lake City Utah USA; ^12^ Department of Population Health University of Kansas School of Medicine Kansas City Kansas USA; ^13^ Siteman Cancer Center, Washington University School of Medicine St. Louis Missouri USA

**Keywords:** colorectal cancer, diet quality, social determinants of health, symptom occurrence

## Abstract

Colorectal cancer (CRC) is a leading cause of cancer mortality in the United States. Many patients with CRC experience physical and psychological symptoms that diminish quality of life and are shaped by social determinants of health (SDOH) and diet quality. This study aimed to (a) identify distinct symptom occurrence subgroups among patients with CRC, and (b) examine differences in SDOH characteristics and diet quality across these symptom occurrence subgroups. We conducted a secondary analysis of the ColoCare Study dataset from the Huntsman Cancer Institute, including CRC patients 6 months postdiagnosis. Latent class analysis identified subgroups based on the presence of symptoms. Chi‐square tests and ANOVA examined subgroup differences in SDOH and diet quality, measured by the Healthy Eating Index (HEI)‐2020. Logistic regression adjusted for cancer stage, comorbidity status, neoadjuvant or adjuvant therapy. Among 256 patients, three subgroups emerged: low (*n* = 84), moderate (*n* = 123), and high symptom occurrence (*n* = 49). Patients with high symptom occurrence were younger (F = 4.48, *p* = 0.01), more often Hispanic (χ² = 9.10, *p* = 0.01), had lower income (χ² = 12.92, *p* = 0.01), and more frequently received neoadjuvant therapy (χ² = 10.30, *p* = 0.006). They reported lower total social support (F = 5.60, *p* = 0.004) and poorer diet quality (F = 9.58, *p* = 0.0001). After adjustment, high symptom occurrence group had lower odds of adequate income (OR = 0.28, *p* = 0.0312), social support (OR = 0.886, *p* = 0.0100), and diet quality (OR = 0.94, *p* = 0.0156). Distinct CRC symptom profiles highlight the need for personalized dietary interventions and enhanced social support to improve symptom management.

**Patient or Public Contribution:**

Findings emphasize care strategies that address SDOH to optimize outcomes and quality of life for patients with CRC.

## 1 Introduction

Colorectal cancer (CRC) is the leading cause of cancer death in the United States (Siegel et al. 2026). In 2026, an estimated 158,850 cases of CRC will be diagnosed in the United States, and an estimated 55,230 deaths for the same year (Siegel et al. 2026). Although survivor rates have improved, many patients who survive CRC face ongoing physical and psychological symptoms (e.g., gastrointestinal symptoms, sexual dysfunction, psychological distress, fatigue, physical and social limitations, insomnia) (Han et al. 2020). The first treatment for CRC is usually surgery, either preceded or followed by chemotherapy and radiation therapy, depending on the disease stage and progression; (American Cancer Society 2023) after the acute side effects of these treatments subside, many survivors report ongoing symptoms such as fatigue, bowel dysfunction, depression, and insomnia (Tantoy et al. 2016). These debilitating symptoms are compounded for patients with advanced stages of cancer or who have comorbidities (Nipp et al. 2017). Patients diagnosed with metastatic CRC or cancer recurrence often experience severe physical and psychological symptoms that significantly reduce quality of life (QoL) and adversely affect cancer survivorship (Griffin‐Sobel 2006; Simmonds 2000).

Inequities in the symptom experiences of patients with CRC are significantly shaped by social determinants of health (SDOH), described as the (a) immediate conditions in which people are born, grow, work, live, and age; and (b) wider set of forces and systems that shape their daily life. (World Health Organization 2010). In line with the heuristic SDOH framework (Thimm‐Kaiser et al. 2023), these influences can be understood through distinct mechanisms, including inequities in SDOH capital (e.g., educational and employment opportunities, income, housing, transportation, food security, and access to affordable, high‐quality health care) and SDOH processes (e.g., social inclusion, nondiscrimination, and other social‐context factors shaping interactions with institutions). These mechanisms may also shape downstream behavioral and environmental exposures, such as diet quality, that can increase or buffer symptom burden. For example, access to affordable high‐quality healthcare services has been linked with better treatment processes and symptom experiences (Islami et al. 2022). Patients with CRC who have a low socioeconomic status, are without health insurance, or who live in rural areas often face challenges in accessing adequate healthcare and treatment (Islami et al. 2022), which can lead to more advanced cancer at diagnosis and more severe symptoms following treatment. These inequities may persist into the survivorship period, shaping long‐term symptom burden and recovery trajectories among CRC survivors.

Diet is a main behavioral and environmental factor affecting CRC symptoms and outcome (Lee et al. 2025). Research suggests that a high‐quality diet has a preventive effect on CRC risk and is associated with a significant reduction in cancer‐related mortality (Schwingshackl et al. 2018). Diet quality indices assess the extent to which individuals’ diets align with established dietary recommendations, with higher scores generally associated with reduced risks of cancer and better overall health outcomes (Alkerwi 2014; Wirt and Collins 2009). A high‐quality diet rich in fruits, vegetables, whole grains, and lean proteins serves not only to reduce CRC risk but to alleviate symptoms and improve response to treatment (Hou et al. 2013).

Identification of associations among SDOH, diet quality, and health outcomes can enhance the development of targeted strategies to improve critical aspects of diet among people living with chronic diseases, including cancer (Wirt and Collins 2009). However, studies on the diet quality of patients with CRC are limited, with most focusing on CRC risk (Drury et al. 2017). To better understand the symptom experiences of patients with CRC, it is important to examine both SDOH and diet quality in relation to symptom subgroups. The aims of this study, therefore, were to (a) identify subgroups of patients with CRC with distinct symptom profiles; and (b) examine differences in SDOH and diet quality for distinct symptom profiles of patients with CRC after adjusting for clinical factors.

### 1.1 Conceptual Framework

**Figure 1:**
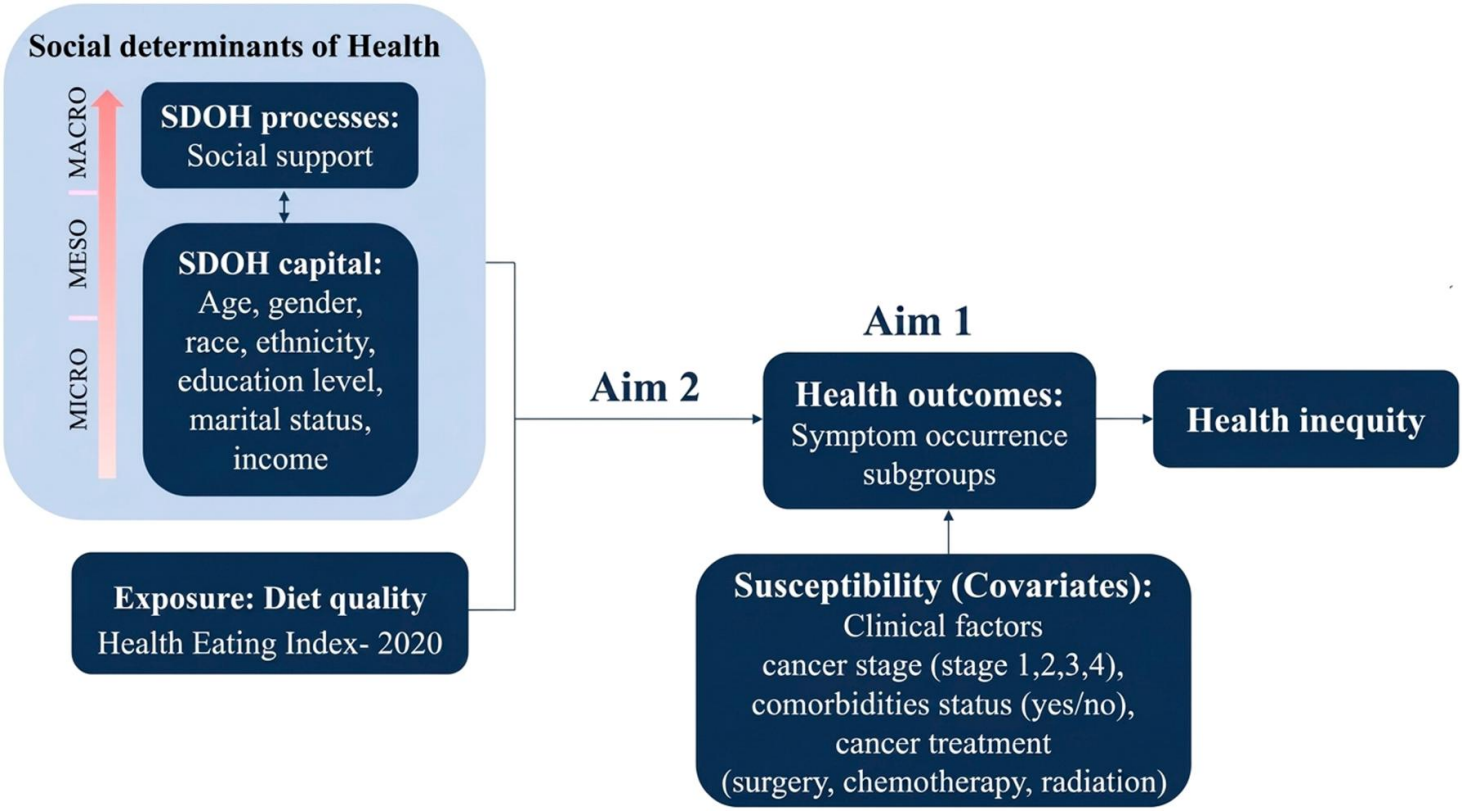
Conceptual framework.

The conceptual framework for this study was guided by the heuristic SDOH framework proposed by (Thimm‐Kaiser et al. 2023), which facilitates the translation of scholarly work on SDOH into evidence‐based interventions aimed at addressing health inequities. Thimm‐Kaiser and colleagues defined and categorized the mechanisms for SDOH as (a) SDOH capital, or quantifiable resources and opportunities that affect health outcomes, for which allotment to individuals or groups is primarily determined by social factors such as experiences shaped by age, gender, race, ethnicity, education level, marital status, income, and living area (rural/urban), among others; and (b) SDOH processes, “or social factors that shape interactions among people, groups, institutions, or systems that impact health” (p. 488) (Thimm‐Kaiser et al. 2023); for the purposes of the current study, our conceptual framework includes social support among SDOH processes. Importantly, consistent with calls to distinguish SDOH from sociodemographic descriptors, age, gender, race, ethnicity, and marital status were treated as demographic characteristics and markers of structural positioning that may shape the distribution of SDOH capital, rather than as SDOH indicators themselves (Lines et al. 2023). According to Thimm‐Kaiser et al. (2023), social capital also includes behavioral and environmental exposures (i.e., “subjection to a health risk or protective factor”) (p. 488); within our framework, diet quality is an exposure that can be a risk or protective factor in influencing the health outcomes of patients with CRC. Lastly, biological susceptibility was described as “the likelihood of morbidity/mortality given the exposure to a health risk factor” (p. 488). In the context of our study, location of cancer, cancer stage, comorbidities, and cancer treatment status were conceptualized as susceptibility and included as key covariates in the analyses. Figure 1 illustrates the conceptual framework according to our study aims.

## 2 Methods

### 2.1 Research Design

The proposed study is a secondary, cross‐sectional data analysis using data from the Colocare Study.

### 2.2 Subject and Setting

The ColoCare Study (ClinicalTrials.gov Identifier: NCT02328677) is a multicenter prospective cohort study of women and men newly diagnosed with primary invasive CRC at any stage; participating U.S. research institutes include Fred Hutchinson Cancer Research Center (Seattle, WA), Moffitt Cancer Center and Research Institute (Tampa, FL), Huntsman Cancer Institute (Salt Lake City, UT), Cedars‐Sinai Medical Center (Los Angeles, CA), Washington University School of Medicine (St. Louis, MI), and the Center for Cancer Research (Memphis, TN) (Ulrich et al. 2019). The dataset includes symptom experiences across multiple time points (3 m, 6 m, 12 m, 24 m, 36 m, 48 m, 60 m), detailed assessments of diet, and other prognostic factors, allowing for a wide range of impactful CRC research. Among the six U.S. cancer centers, only three had complete data on SDOH, diet quality, and symptom experiences at the 6‐month time point following CRC diagnosis. Therefore, only data from these three centers were included in the analysis for this study.

To better understand the distinct characteristics of SDOH and diet quality across different symptom groups among patients with CRC, we analyzed symptom experience data collected at the same 6‐month post‐diagnosis time point: The Food Frequency Questionnaire (FFQ), collected 6 months after diagnosis, comprises a defined list of foods and beverages, with response categories that capture the usual frequency of consumption over a specified time period (National Cancer Institute 2024a). Additionally, social support variables were collected 6 months after a CRC diagnosis.

### 2.3 Study Measures

#### 2.3.1 Symptom Experiences

Cancer symptom variables were assessed using the European Organization for Research and Treatment of Cancer Quality of Life Questionnaire Core‐30 (EORTC‐QLQ‐C30), a 30‐item measure designed to be cancer‐specific, multidimensional in structure, appropriate for self‐administration, and applicable across a range of cultural settings (Fayers and Bottomley 2002). The EORTC QLQ‐C30 assesses health‐related QoL of patients with cancer using five functional scales (physical, role, emotional, social, and cognitive), nine symptom scales (fatigue, nausea/vomiting, pain, dyspnea, appetite loss, insomnia, constipation, diarrhea, financial difficulties), and a global health status/QoL scale (EORTC Data Center 2018). For this study, we analyzed nine symptoms (fatigue, nausea, vomiting, pain, dyspnea, appetite loss, insomnia, constipation, and diarrhea), excluding the financial difficulties item; nausea and vomiting were treated as separate items from the nausea/vomiting symptom measure. Each symptom item was assessed on a 4‐point Likert scale (1 = not at all; 4 = very much) and analyzed separately as an individual categorical variable to identify distinct symptom patterns, not summed into an overall symptom severity score (EORTC Data Center 2018). Symptom severity was originally categorized into four levels (“not at all,” “a little,” “quite a bit,” and “very much”); however, responses were highly skewed toward “not at all” and “a little.” To address this imbalance and simplify the analysis, symptom responses were dichotomized into “no symptoms (symptom absence)” (“not at all”) and “having some symptoms (symptom presence)” (“a little,” “quite a bit,” and “very much”) (see Table 1).

**Table 1:** Descriptions of measures used for analysis.

Construct	Descriptions		Range & interpretation of scores
Symptom experiences (12 months after diagnosis)	Fatigue (3 items)		Total score range = 3–12 (higher scores indicate worse symptom experience)
	Nausea/vomiting (2 items)		Total score range = 2–8 (higher scores indicate worse symptom experience)
	Pain (2 items)		Total score range = 2–8 (higher scores indicate worse symptom experience)
	Dyspnea (1 item)		Total score range = 1–4 (higher scores indicate worse symptom experience)
	Appetite loss (1 item)		Total score range = 1–4 (higher scores indicate worse symptom experience)
	Insomnia (1item)		Total score range = 1–4 (higher scores indicate worse symptom experience)
	Constipation (1item)		Total score range = 1–4 (higher scores indicate worse symptom experience)
	Diarrhea (1item)		Total score range = 1–4 (higher scores indicate worse symptom experience)
Social determinants of health (SDOH) (Baseline)	SDOH capital	Sociodemographic	Age, gender, education attainment, marital status/living situation
		Socioeconomic status	Income
		Social identity	Race, ethnicity
	SDOH process	Social support	ENRICHD Social Support Instrument (ESSI) (7 items) Total score range = 0–35 (higher scores indicate higher perceived social support)
Diet quality (6 months after diagnosis)	Diet quality	Health eating index‐2020 (HEI‐2020) *Adequacy components (9 items): Total Fruits, Whole Fruits, Total Vegetables, Greens and Beans, Whole Grains, Dairy, Total Protein Foods, Seafood and Plant Proteins, and Fatty Acids, which is a ratio of unsaturated versus saturated fatty acids*. *Moderation components: (4 items) Refined Grains, Sodium, Added Sugars, and Saturated Fats*	Total score range = 0–100 (higher scores indicate higher diet quality)
Susceptibility	Disease and health variables		Location of cancer, cancer stage, comorbidities, cancer treatment, cancer recurrence status

#### 2.3.2 Social Determinants of Health

SDOH were assessed by SDOH capital and SDOH processes based on a revision of Thimm‐Kaiser et al.'s Center for Latino Adolescent and Family Health Framework (Thimm‐Kaiser et al. 2023) (see Figure 1). To represent SDOH capital, sociodemographic (age, gender, education level, marital status/living situation), SES (income), and social identity including race and ethnicity were measured. For SDOH process, social support was measured using the ENRICHD social support instrument (ESSI). ESSI uses a 5‐item Likert format ranging from 1 = *none of the time* to 5 = *all of the time*. Items were designed to measure various types of support including perceived emotional support, instrumental support, appraisal support, and marital status (Cronbach's α = 0.88) (Mitchell et al. 2003). Higher scores indicated higher perceived social support (see Table 1).

#### 2.3.3 Diet Quality

Diet quality indices assess the extent to which an individual's diet aligns with established dietary recommendations (Wirt and Collins 2009). Diet quality was measured using the HEI‐2020 (Healthy Eating Index), a tool that assesses how well a set of food components aligns with the *Dietary Guidelines for Americans, 2020–2025* edition (U.S. Department of Agriculture 2020). HEI‐2020 is made up of 13 components comprising foods to consume more of, called adequacy components (i.e., Total fruits, Whole fruits, Total vegetables, Greens and beans, Whole grains, Dairy, Total protein foods, Seafood and plant proteins, Fatty acids), and foods to consume less of, called moderation components (i.e., Refined grains, Sodium, Added sugars, and Saturated fats). For adequacy components, higher scores reflect higher intake; for moderation components, higher scores reflect lower intake. Components are scored on a density basis (per 1000 calories), with the exception of the fatty acids’ component, which is scored as a ratio of unsaturated to saturated fatty acids. Each component is assigned a standard for achieving the maximum score based on consuming the appropriate amount, then the component scores are summed to achieve the total HEI score, which indicates overall diet quality; a higher HEI score indicates a healthier diet. A total score of 100 represents perfect alignment with the dietary guidelines, and lower scores indicate less adherence (U.S. Department of Agriculture 2023) (see Table 1).

#### 2.3.4 Susceptibility

Susceptibility comprised disease and health variables including location of cancer, cancer stage, comorbidities, cancer treatment, and cancer recurrence status, which served as covariates/control variables. Locations of cancer were the colon and rectum. Cancer stage was divided into stage Ⅰ, stage Ⅱ, stage Ⅲ, and stage Ⅳ. The comorbidity status was dichotomized into two categories: those with at least one comorbidity (arthritis, asthma, Crohn's, diabetes, Familial Adenomatous Polyposis (FAP), Irritable Bowel Disease (IBD), Hashimoto's disease, Grave's disease, heart diseases, history of colon polyps/adenomas, hyperlipidemia, hypertension, lynch Syndrome, lupus, stroke, stomach ulcer, ulcerative colitis) and those without. Cancer treatment included surgery, chemotherapy, radiation therapy, and chemoradiation. Lastly, cancer recurrence status was included (see Table 1).

### 2.4 Statistical Analyses

Descriptive statistics were used to detail the sample characteristics for the estimated sample size (*N* = 256) and all study measures. The details of the sample size determination were described in Supporting Information S1: Table 1. Nondirectional inferential statistical tests were conducted at a 0.05 significance level, which was not adjusted for multiple tests due to the exploratory nature of the proposed study. Effect sizes and 95% confidence interval were reported to address clinical significance. Data analyses were completed using R software 4.4.1 version (R core Team, Auckland, New Zealand).

#### 2.4.1 Identify Subgroups of Patients With CRC With Distinct Symptom Profiles

Latent class analysis (LCA) was used to identify subgroups of patients with CRC with different symptom experiences based on nine symptom categories (fatigue, nausea, vomiting, pain, dyspnea, appetite loss, insomnia, constipation, diarrhea) (EORTC Data Center 2018). LCA is a person‐centered modeling technique used to identify latent subgroups (classes) based on an unobserved construct (Lanza and Rhoades 2013). Multiple considerations determined the final optimal model: First, the goodness‐of‐fit of the model was evaluated with the Akaike Information Criterion (AIC) and Bayesian Information Criterion (BIC), sample‐adjusted Bayesian Information criterion (aBIC), and Bootstrap Likelihood Ratio Test (BLRT) (Weller et al. 2020). Second, the clinical meaningfulness and interpretability of the model and sample sizes within each subgroup were examined. If a sample size within any subgroup was less than 25, the model was rejected due to limited practical meaningfulness (Berlin et al. 2014; Lubke and Neale 2006). Third, entropy was calculated to access the accurate classification of the final model (Celeux and Soromenho 1996). Entropy value ranges from 0 to 1, with a higher value indicating a more accurate classification or separation of classes (Celeux and Soromenho 1996). Although there is no established cut‐off, entropy greater than 0.8 was considered acceptable (Weller et al. 2020).

#### 2.4.2 Examine the Differences in SDOH and Diet Quality for Distinct Symptom Profiles in Patients With CRC After Adjusting for Clinical Factors

Bivariate and multiple regression models were used to determine associations between each individual SDOH variable, diet quality score, and distinct cancer symptom profiles identified. First, a bivariate analysis was conducted to examine the unadjusted association between each individual SDOH (independent variables), HEI‐2020 score (National Cancer Institute 2024b) (independent variables), and distinct cancer symptom profiles (categorical dependent variable). For continuous variables, social support, and diet quality, an Analysis of Variance (ANOVA) was conducted, and Cohen's F was reported to assess effect sizes. When significant differences were detected by ANOVA, Tukey's test was applied for post hoc comparisons. Chi‐square tests were conducted for categorical variables including sociodemographic (age, gender, education level, marital status), socioeconomic status (income), social identity (race and ethnicity), and odds ratio was calculated to assess effect size. Next, multiple multinomial logistic regression analysis was used adjusting for important confounders, including location of cancer, cancer stage, comorbidities, cancer treatment, and cancer recurrence status. Adjusted odds ratios and 95% confidence intervals were reported. Clinical significance was addressed based on the results. In addition, radar plots for each of the 13 HEI‐2020 components (i.e., total fruits, whole fruits, total vegetables, greens and beans, whole grains, dairy, total protein foods, seafood and plant proteins, fatty acids, refined grains, sodium, added sugars, and saturated fats) are presented by different symptom profiles.

## 3 Results

### 3.1 Sample Characteristics

Table 2 presents the descriptive statistics of sample characteristics. The study sample consisted of 256 participants, with a mean age of 59.33 years (SD = 13.74). Participants were recruited from the Huntsman cancer center (53%), Washington University cancer center (24%), and Cedars‐Sinai Medical Center (23%). The sample was predominantly male (53%) and non‐Hispanic White (80%), with 14% identifying as non‐Hispanic Black/African American and 11% identifying as Hispanic/Latino. Education attainment varied, with 74.25% reporting a college or graduate‐level education, and 25% having completed grade school or some high school. Among participants, 65% were married and household income levels varied, with 54% earning $70,001 to $150,000 annually, and 24% earning $30,000 or less. Clinically, half of participants had advanced stages of cancer (52%), and 78% reported comorbidities. Most participants received surgery (95%), 42% received adjuvant chemotherapy, and 35% received neoadjuvant chemotherapy. Social support was rated highly, with a mean total score of 25.76 (SD = 4.91), and the median values for all subscales suggested robust support networks. Dietary quality data, available for 137 participants due to limited data availability, revealed a mean total diet quality score of 60.57 (SD = 11.41). Median scores highlighted areas of better adherence to dietary recommendations, particularly for vegetables, seafood, and plant proteins. Conversely, lower adherence was observed for refined grains and added sugars.

**Table 2:** Sample characteristics.

Categories	Total Sample (*N* = 256)	
**SDOH capital factors**	***N (%)***	
**Current age**	256	
Less than 30 years old	3 (1)	
Age 30–39	10 (4)	
Age 40–49	61 (24)	
Age 50–59	59 (23)	
More than 60 years old	123 (48)	
**Current age (continuous)**	**Mean ± SD**	
	59.33 + 13.74	
**Cancer center**	256	
Huntsman Cancer Center, Salt Lake City	135 (53)	
Washington University, St. Louis	61 (24)	
Cedars‐Sinai Medical Center, Los Angeles	60 (23)	
**Gender**	256	
Male	136 (53)	
Female	120 (47)	
**Race**	256	
NH White	204 (80)	
NH Black/AA	35 (14)	
Other	17 (6)	
**Ethnicity**	256	
Non‐Hispanic/Latino	229 (89)	
Hispanic/Latino	27 (11)	
**Education Attainment**	202	
Grade school or high school	52 (26)	
Undergraduate college	113 (56)	
Graduate or professional school	37 (18)	
**Marital status**	202	
Married	130 (65)	
Divorced, separated, or widowed	35 (17)	
Single or Unmarried	37 (18)	
**Income**	170	
$30,000 or less	41 (24)	
$30,001–$69,999	38 (22)	
$70,000–$149,999	56 (33)	
More than $150,000	35 (21)	
**Clinical factors**	N (%)	
**Cancer stage**	243	
Stage 1	41 (17)	
Stage 2	76 (31)	
Stage 3	100 (41)	
Stage 4	26 (11)	
**Comorbidities status**	228	
Yes (I have a diseases along with cancer)	178 (78)	
No (I do not have a diseases rather than cancer)	50 (22)	
**Having surgery**	186	
Yes	176 (95)	
No	10 (5)	
**Adjuvant therapy**	219	
Yes‐ Chemotherapy, Radiochemotherapy	93 (42)	
No	126 (58)	
**Neoadjuvant therapy**	227	
Yes‐ Chemotherapy, Radiotherapy, Radiochemotherapy	79 (35)	
No	148 (65)	

**Social support (*n*** = **256)**	**Mean (SD)**	**Median (25th–75th percentile)**
Total social support (range: 5–30) (*n* = 251)	25.76 (4.91)	27.00 (23.00–30.00)
Listening (range: 1–5) (*n* = 252)	4.49 (0.80)	5.00 (4.00–5.00)
Advice (range: 1–5) (*n* = 252)	4.38 (0.91)	5.00 (4.00–5.00)
Love (range: 1–5) (*n* = 256)	4.47 (1.03)	5.00 (4.00–5.00)
Chores (range: 1–5) (*n* = 256)	3.84 (1.34)	4.00 (3.00–5.00)
Emotional support (range: 1–5) (*n* = 251)	4.36 (1.04)	5.00 (4.00–5.00)
Confidant (range: 1–5) (*n* = 256)	4.25 (1.06)	5.00 (4.00–5.00)

**Diet quality (*n*** = **137)**	**Mean (SD)**	**Median (25th–75th percentile)**
Total vegetables (range: 0–5)	3.81 (1.19)	4.01 (3.16–5.00)
Greens and beans (range: 0–5)	3.48 (1.65)	4.3 (1.76–5.00)
Total fruits (range: 0–5)	2.71 (1.77)	2.50 (1.03–4.56)
Whole fruits (range: 0–5)	2.87 (1.77)	2.86 (1.46–4.91)
Whole grains (range: 0–10)	3.74 (2.82)	3.04 (1.50–5.85)
Total diary (range: 0–10)	5.37 (2.52)	5.14 (3.25–7.21)
Total protein (range: 0–5)	4.78 (0.57)	5.00 (5.00–5.00)
Seafood and plant proteins (range: 0–5)	4.23 (1.26)	5.00 (4.06–5.00)
Fatty acids (range: 0–10)	5.03 (2.59)	5.21 (3.12–6.53)
Sodium (range: 0–10)	4.03 (2.56)	3.69 (2.13–5.41)
Refined grains (range: 0–10)	8.56 (2.60)	10.00 (7.95–10.00)
Added sugars (range: 0–10)	6.79 (2.85)	6.99 (5.28–8.91)
Saturated fats (range: 0–10)	5.16 (3.15)	5.62 (2.66–7.45)
Total diet quality (range: 0–100)	60.57 (11.41)	60.21 (53.17–68.55)

### 3.2 Symptom Occurrence Subgroups

Based on fit indices for the 2‐ to 5‐class latent class analysis (LCA) models (details in Appendix SA), the best model fit was selected as a 3‐class model (i.e., high, moderate, low), despite the 4‐class model having the lowest Akaike's Information Criterion (AIC) and Bayesian Information Criterion (BIC) values, as the 3‐class solution was better supported by theoretical evidence. The 3‐class model also exhibited lower Consistent Akaike Information Criterion (CAIC) values than the 4‐class model and had the same entropy (0.77).

**Figure 2:**
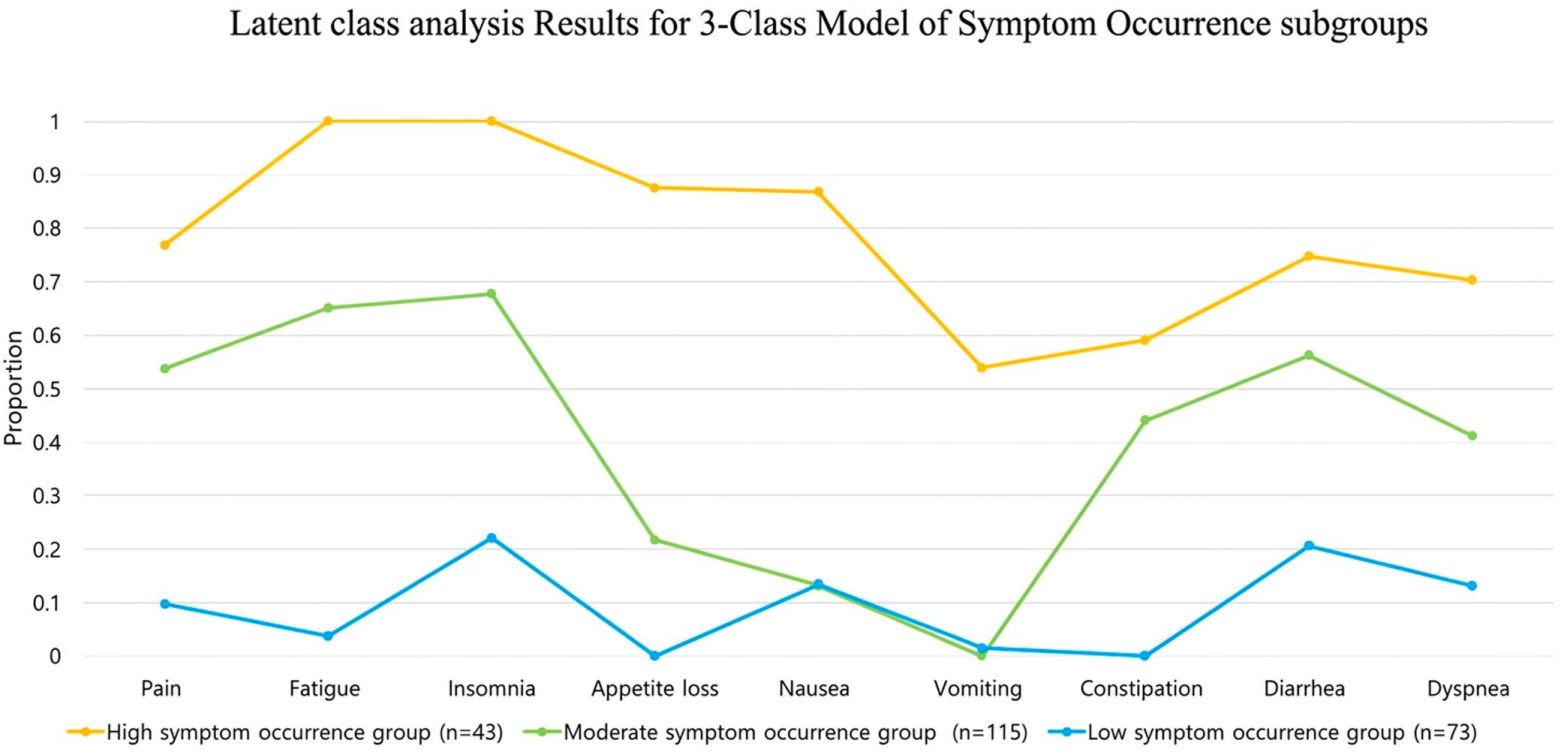
LCA results for 3‐class model of symptom occurrence subgroups.

As shown in Figure 2, the three classes were named based on the occurrence of the nine CRC symptoms. The low class consisted of 32.8% (*n* = 84) of the sample, who reported relatively low symptom occurrence rates for most of the symptoms. The moderate class was the largest and consisted of 48.0% (*n* = 123) of the sample, who reported occurrence of moderate number of symptoms for nine CRC symptoms; nausea and vomiting were also minimal in this symptom group. The high class was the smallest and consisted of 19.1% (*n* = 49) of the sample, who reported occurrence of a high number of symptom rates for the nine CRC symptoms.

### 3.3 Individual Characteristics of Symptom Occurrence Subgroups

There were statistically significant differences (*p* < 0.05) in number of symptoms, SDOH factors (ethnicity, income), clinical factors (cancer stage, neoadjuvant therapy), social support (listening, advice, love, chores, emotional support, total social support), and diet quality (total vegetables, greens and beans, whole fruit, whole grains, refined grains, added sugar, total diet quality) between subgroups (Table 3). The high symptom occurrence group experienced seven symptoms, the moderate 3.6, and the low 0.9. The characteristics reported by the high symptom occurrence group (Class 3) included unmarried status (44.12%), the lowest percentage of college education or graduate school education (64.71%), income over $30,000 (58.82%), the highest percentage of over Stage 3 cancer (62.50%), and receiving neoadjuvant therapy (53.33%). Additionally, the high symptom occurrence group reported the lowest total social support score (23.90 ± 6.61), including all social support subscores, as well as the lowest total diet quality score (54.42 ± 13.85), encompassing all diet quality subscores (Table 3). In addition, moderate effect sizes (Cramer's V > 0.1) were observed for ethnicity, income, neoadjuvant therapy, and added sugar in diet quality.

**Table 3:** Individual characteristics of symptom occurrence subgroups.

	Class 1 (High symptom occurrence) (*n* = 49)	Class 2 (Moderate symptom occurrence) (*n* = 123)	Class 3 (Low symptom occurrence) (*n* = 84)	Effect size	*p* value
	*Mean* (*SD)*	*Mean* (*SD)*	*Mean* (*SD)*	Cohens’ F	F (*p* value)
Number of symptoms	7.06 (1.25)	3.64 (1.34)	0.87 (0.74)	0.7802	449.05 **(** < **.0001)**

**SDOH**	***Mean (SD)***	***Mean (SD)***	***Mean (SD)***	Cohens’ F	
Current age	54.80 **(**11.85)	61.55 (15.15)	58.71 (11.90)	0.0342	4.48 **(0.0122)**
Age at diagnosis	51.59 **(**11.60)	58.42 (14.93)	54.94 (11.42)	0.0383	5.03 **(0.0072)**

	***N* (%)**	***N* (%)**	***N* (%)**	Cramer's V	**X** ^**2**^ ***(p‐value)***
**Gender**	49	123	84		
Male	30 (61.22)	70 (56.91)	36 (42.86)	0.1473*	5.5550 (0.0622)
Female	19 (38.78)	53 (43.09)	48 (57.14)		
**Race**	49	123	84		
White, NH	41 (83.67)	100 (81.30)	63 (75.00)	0.0843	1.8190 (0.4027)
Others	8 (16.33)	23 (18.70)	21 (25.00)		
**Ethnicity**	49	123	84		
Non‐Hispanic	43 (87.76)	117 (95.12)	69 (82.14)	0.1885*	9.097 (**0.0106**)
Hispanic	6 (12.24)	6 (4.88)	15 (17.86)		
**Marital status**	34	104	64		
Married	19 (55.88)	73 (70.19)	38 (59.38)	0.1278*	3.3008 (0.1920)
Unmarried	15 (44.12)	31 (29.81)	26 (40.63)		
**Education**	34	104	64		
Grade school or some high school	12 (35.29)	25 (24.04)	15 (23.44)	0.0985	1.9586 (0.3756)
College or graduate school	22 (64.71)	79 (75.96)	49 (76.56)		
**Income**	34	85	51		
Less than $30,000	14 (41.18)	16 (18.82)	11 (21.57)	0.2013*	6.8891 (**0.0319**)
$30,000 or more	20 (58.82)	69 (81.18)	40 (78.43)		
**Clinical factors**	N (%)	N (%)	N (%)		
**Cancer stage**	48	116	79		
Stage 1	7 (14.58)	23 (19.83)	11 (13.92)	0.1979*	19.0261 (**0.0041**)
Stage 2	11 (22.92)	26 (22.41)	39 (49.37)		
Stage 3	24 (50.00)	52 (44.83)	24 (30.38)		
Stage 4	6 (12.50)	15 (12.93)	5 (6.33)		
**Comorbidities**	41	109	78		
No	33 (80.49)	88 (80.37)	57 (73.08)	0.0870	1.7276 (0.4216)
Yes	8 (19.51)	21 (19.27)	21 (26.92)		
**Neoadjuvant Therapy**	45	105	77		
No	21 (46.67)	69 (65.71)	58 (75.32)	0.2130*	10.3031 (**0.0058**)
Yes	24 (53.33)	36 (34.29)	19 (24.68)		
**Adjuvant Therapy**	44	99	76		
No	28 (63.64)	57 (57.58)	41 (53.95)	0.0699	1.0709 (0.5854)
Yes	16 (36.36)	42 (42.42)	35 (46.05)		

	***Mean (SD)***	***Mean (SD)***	***Mean (SD)***	Cohens’ F	**F (*p*‐value)**
**Social support**	49	123	84		
Total social support	23.90 **(**6.61)	25.76 (4.87)	26.86 (3.27)	0.0424	5.60 (**0.0042**)
Listening (range: 1–5)	4.31 (0.89)	4.43 (0.89)	4.68 (0.54)	0.0316	4.07 **(0.0183**)
Advice (range: 1–5)	4.24 (0.80)	4.23 (1.08)	4.67 (0.59)	0.0511	6.70 (**0.0015**)
Love (range: 1–5)	4.14 (1.41)	4.61 (0.76)	4.46 (1.08)	0.0281	3.66 **(0.0270)**
Chores (range: 1–5)	3.37 (1.55)	3.91 (1.25)	4.01 (1.28)	0.0306	4.00 **(0.0195**)
Emotional support (range: 1–5)	3.90 (1.34)	4.36 (0.99)	4.64 (1.08)	0.0636	8.43 (**0.0003)**
Confidant (range: 1–5)	3.94 (1.39)	4.27 (0.98)	4.39 (0.92)	0.0226	2.93 (0.0555)

	***Mean (SD)***	***Mean (SD)***	***Mean (SD)***	**Cohen's F**	**F (*p*‐value)**
	High symptom occurrence	Moderate symptom occurrence	Low symptom occurrence		
**Diet quality**	27	67	43		
Total vegetables	3.14 (1.42)	3.93 (1.10)	4.05 (1.03)	0.0810	5.90 (**0.0035)**
Greens and beans	2.42 (1.61)	3.75 (1.50)	3.73 (1.65)	0.1035	7.74 **(0.0007**)
Total fruits	2.05 (1.72)	2.60 (1.59)	3.31 (1.94)	0.0651	4.66 (**0.0110**)
Whole fruits	2.24 (1.61)	2.84 (1.77)	3.32 (1.81)	0.0463	3.25 **(0.0417)**
Whole grains	3.39 (2.87)	3.25 (2.51)	4.74 (3.05)	0.0575	4.09 **(0.0189)**
Total diary	5.74 (1.74)	5.13 (2.58)	5.52 (2.85)	0.0098	0.67 (0.5153)
Total protein	4.69 (0.43)	4.82 (0.69)	4.78 (0.44)	0.0084	0.57 (0.5680)
Seafood and plant proteins	4.03 (1.16)	4.37 (1.30)	4.13 (1.27)	0.0125	0.85 (0.4297)
Fatty acids	4.46 (3.38)	4.93 (2.42)	5.55 (2.23)	0.0230	1.58 (0.2097)
Sodium	5.03 (3.17)	3.75 (2.40)	3.83 (2.57)	0.0354	2.46 (0.0895)
Refined grains	8.14 (3.63)	8.20 (2.59)	9.38 (1.49)	0.0460	3.23 (**0.0426)**
Added sugars	4.36 (3.41)	7.41 (2.03)	7.35 (2.83)	0.1794*	14.65 **(** < **.0001)**
Saturated fats	4.74 (3.94)	4.85 (2.66)	5.91 (3.25)	0.0265	1.83 (0.1649)
Total diet quality	54.42 (13.85)	59.82 (8.15)	65.60 (11.63)	0.1251*	9.58 (**0.0001)**

*Note:* Bold values = *p* < 0.05; * = moderate effect size (Cramér's V > 0.10).

**Figure 3:**
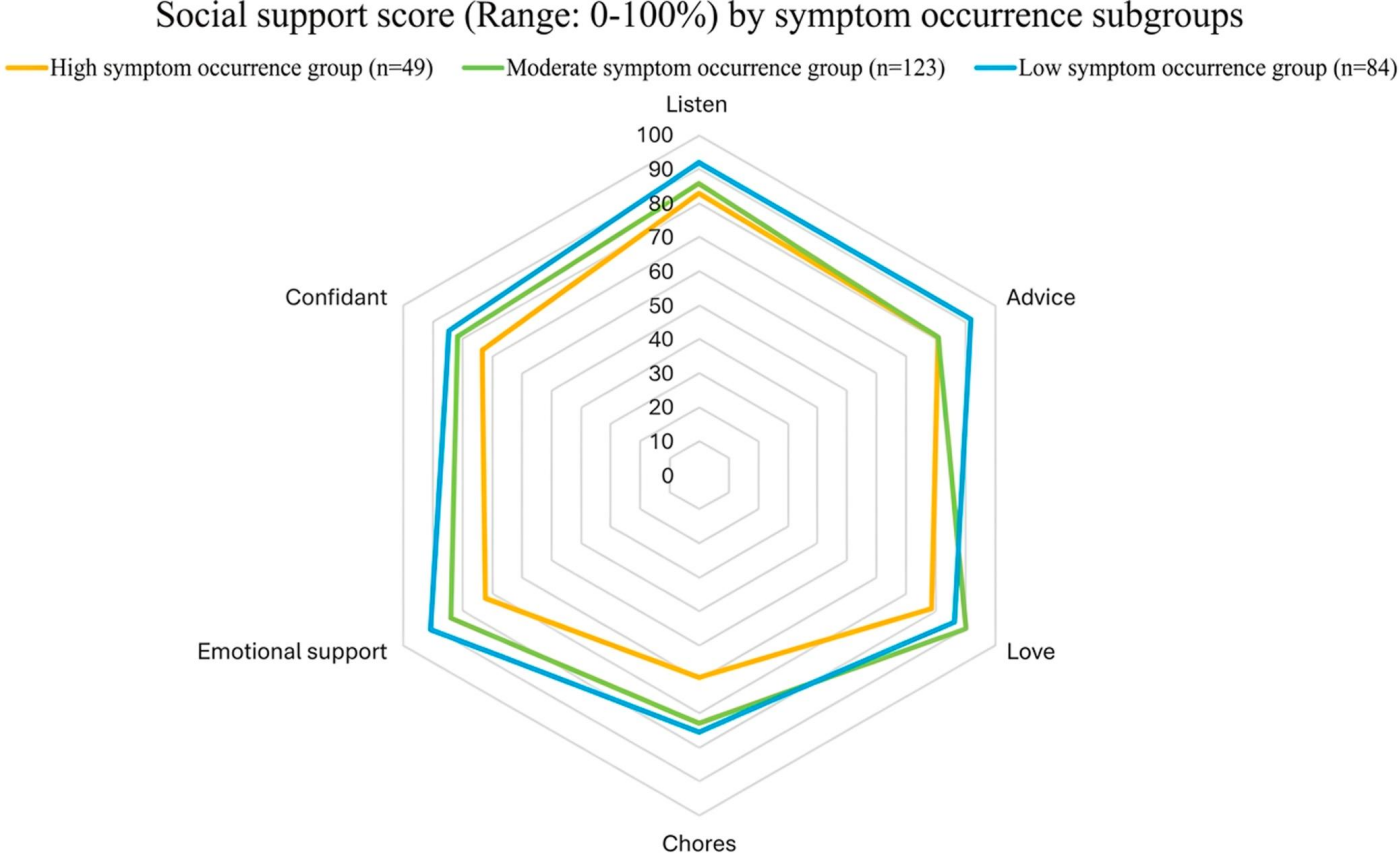
Social support score (range: 0%–100%) by symptom occurrence subgroups.

**Figure 4:**
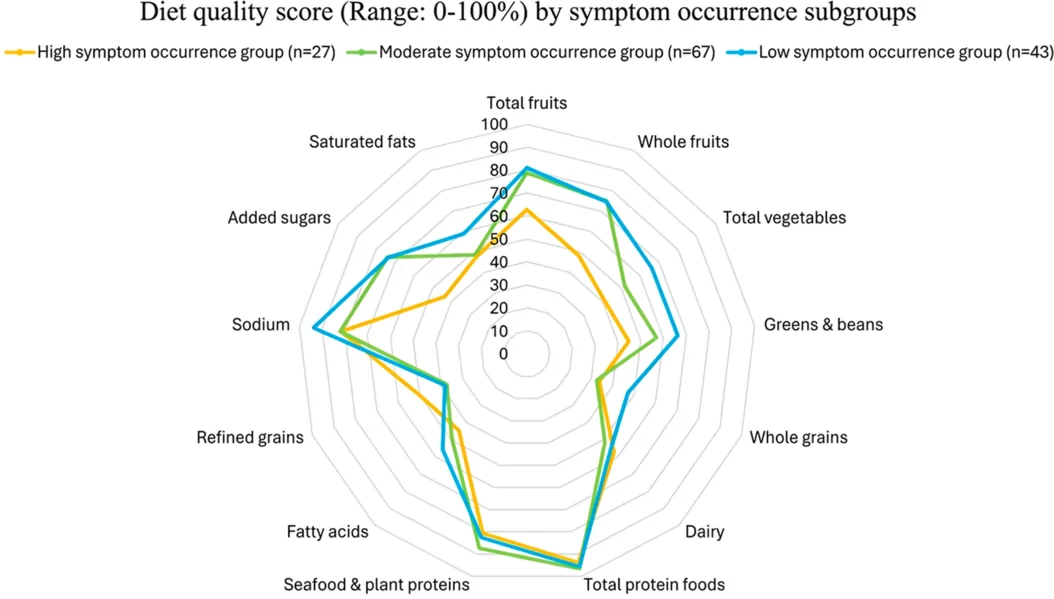
Diet quality score (range: 0%–100%) by symptom occurrence subgroups.

A radar plot (Figures 3 and 4) presented the components of social support and diet quality simultaneously. For diet quality, the radar plot distinguishes between nutrition components with score ranges of 0–5 (total vegetables, greens and beans, total fruits, whole fruits, total protein, and seafood and plant proteins) and those with score ranges of 0–10 (whole grains, total dairy, fatty acids, sodium, refined grains, added sugars, and saturated fats). As the component scores had different ranges (0–5 vs. 0–10), for the radar plot, scores were converted to percentages (0%–100%) by dividing by the maximum possible score and multiplying by 100. The plot illustrates that participants in the high symptom occurrence group consistently exhibit lower diet quality component scores compared to those in the low symptom occurrence group, who demonstrate higher component scores. Among the dietary components, the most pronounced difference was observed in added sugar intake.

### 3.4 Adjusted Associations in Symptom Occurrence Subgroups

Tables 4 and 5 present adjusted odds ratio (OR) estimates, along with their corresponding 95% confidence intervals (CIs) and *p*‐values, for associations between prespecified SDOH indicators and diet quality measures and symptom profile comparison groups.

**Table 4:** Adjusted association between SDOH and symptom occurrence subgroups using multinominal regression analysis.

	High symptom occurrence group (*n* = 84) versus Low symptom occurrence group (*n* = 49)		Moderate symptom occurrence group (*n* = 123) versus Low symptom occurrence group (*n* = 49)	
	aOR^a^ (95% CI)	*p* value	aOR^a^ (95% CI)	*p* value
**Income**				
Over $30,000 versus $ Less than 30,000 (ref)	0.28 (0.09–0.89)	**0.0312**	1.52 (0.58–3.98)	0.3981
**Ethnicity**				
Hispanic versus non_Hispanic (ref)	0.50 (0.05–4.99)	0.5555	0.39 (0.07–2.20)	0.2868
**Social support**				
Listening	0.50 (0.27–0.90)	**0.0216**	0.68 (0.40–1.17)	0.1656
Advice	0.50 (0.28–0.88)	**0.0167**	0.55 (0.34–0.90)	**0.0177**
Love	0.82 (0.57–1.18)	0.2774	1.27 (0.88–1.84)	0.2002
Chores	0.75 (0.53–1.07)	0.1123	1.06 (0.79–1.42)	0.7025
Emotional support	0.31 (0.18–0.55)	**< 0.0001**	0.55 (0.33–0.92)	**0.0229**
Total social support	0.87 (0.81–0.97)	**0.0100**	0.96 (0.88–1.04)	0.3283

*Note:* Bold values indicate = *p* < 0.05.

^a^ Adjusted covariates: cancer stage, comorbidities status, receiving adjuvant chemotherapy, receiving neoadjuvant chemotherapy.

**Table 5:** Adjusted association between diet quality and symptom occurrence subgroups using multinominal regression analysis.

	High symptom occurrence group (*n* = 27) versus Low symptom occurrence group (*n* = 43)		Moderate symptom occurrence group (*n* = 67) versus Low symptom occurrence group (*n* = 43)	
	aOR^a^ (95% CI)	*p* value	aOR^a^ (95% CI)	*p* value
**Diet quality**				
Total vegetable	0.62 (0.39–0.10)	**0.0481**	1.04 (0.71–1.53)	0.8267
Greens and beans	0.74 (0.52–1.05)	0.0942	1.14 (0.86–1.51)	0.3561
Total fruit	0.79 (0.51–1.24)	0.3059	1.11 (0.80–1.55)	0.5345
Whole fruit	0.79 (0.56–1.10)	0.1637	0.98 (0.76–1.27)	0.8956
Whole grain	0.10 (0.80–1.24)	0.9881	0.93 (0.78–1.19)	0.4181
Total diary	0.93 (0.73–1.17)	0.5287	0.85 (0.71–1.02)	0.0720
Total protein	1.09 (0.50–2.37)	0.8301	1.65 (0.67–4.07)	0.2806
Seafood and plant proteins	1.11 (0.73–1.69)	0.6291	1.33 (0.92–1.91)	0.1287
Fatty acids	0.91 (0.74–1.19)	0.3572	0.97 (0.83–1.14)	0.7319
Sodium	1.19 (0.96–1.48)	0.1069	0.91 (0.77–1.10)	0.3450
Refined grain	0.81 (0.63–1.05)	0.1153	0.81 (0.65–1.05)	0.0696
Added sugar	0.65 (0.51–0.83)	**0.0004**	1.13 (0.94–1.36)	0.2034
Total diet quality	0.94 (0.89–0.99)	**0.0230**	0.98 (0.94–1.02)	0.3443

*Note:* Bold values indicate = *p* < 0.05.

^a^ Adjusted covariates: cancer stage, comorbidities status, receiving adjuvant chemotherapy, receiving neoadjuvant chemotherapy.

After adjusting for clinical covariates (i.e., susceptibility: stage of cancer, comorbidity status, and receipt of neoadjuvant or adjuvant therapy), an increase in one unit of income (> $30,000 vs. < $30,000) was associated with reduced odds of being in the high symptom occurrence group compared to the low symptom occurrence group(aOR = 0.279, *p* = 0.0312). In the moderate symptom occurrence group (Typology 2), an increase in one unit of social support was associated with reduced odds of being in the moderate symptom occurrence group compared to the low symptom occurrence group, including advice (aOR = 0.551, *p* = 0.0177) and emotional support (aOR = 0.550, *p* = 0.0229). In the high symptom occurrence group (Typology 3), an increase in one unit of social support was associated with reduced odds of being in the high symptom occurrence group compared to the low symptom occurrence group, including listening support (aOR = 0.497, *p* = 0.0216), advice (aOR = 0.496, *p* = 0.0167), emotional support (aOR = 0.312, *p* < 0.0001), and total social support (OR = 0.886, 95% CI: 0.808–0.972, *p* = 0.0100) (Table 4).

Diet quality was also associated with significantly reduced odds of being in the high symptom occurrence group. Specifically, an increase in total vegetable intake was associated with reduced odds of being in the high symptom occurrence group compared to the low symptom occurrence group (aOR = 0.622, *p* = 0.0481). Higher HEI added sugars component scores were associated with lower odds of being in the high symptom occurrence group versus the low symptom occurrence group (aOR = 0.65, *p* = 0.0004), indicating that better adherence to the recommendation to limit added sugars was associated with a lower likelihood of high symptom occurrence. Higher total diet quality scores were associated with lower odds of being in the high symptom occurrence group compared to the low symptom occurrence group (aOR = 0.94, *p* = 0.0230). No significant differences were observed between the moderate and low symptom occurrence groups after adjusting for clinical covariates (Table 5).

## 4 Discussion

This study explores the relationships between SDOH and symptom occurrence, as well as diet quality and symptom occurrence, in patients with CRC. To our knowledge, this is the first study to examine how specific dietary components are associated with symptom occurrence subgroups of patients with CRC. By considering the contextual influences of SDOH and diet quality through exposure to health risks or protective factors, this study provides new insights into their impact on symptom experiences after controlling for clinical factors. Several symptom occurrence groups were identified based on nine symptom components: fatigue, nausea, vomiting, pain, dyspnea, appetite loss, insomnia, constipation, and diarrhea. Prior research has examined symptoms and associated quality of life based on subgroups (Gigic et al. 2018), and multiple co‐occurring symptom profiles among patients with gastrointestinal cancers (Lin et al. 2021). Our findings aligned with previous research, in that approximately 10% of patients with CRC reported experiencing fatigue, pain, insomnia, and diarrhea. In contrast, fewer than 10% reported symptoms such as dyspnea, nausea, vomiting, loss of appetite, or constipation (Glare et al. 2014; Han et al. 2020; Lin et al. 2023; Lin et al. 2022).

Contrary to what we hypothesized, individuals of Hispanic ethnicity, a racial and ethnic minoritized group, were less likely to have high symptom occurrence, possibly due to underreporting of symptoms or higher rates of social desirability bias (Hopwood et al. 2009). This unexpected finding may be explained by the “Hispanic paradox,” an epidemiological phenomenon whereby Latino people in the United States exhibit better health outcomes than other demographic groups, including non‐Hispanic White people, despite having greater risk factors such as lower socioeconomic status and limited access to healthcare (Gonzalez‐Guarda and Pearson 2024). However, the paradox is not universal: For cancers with 5‐year observed survival rates 50% and higher, the Hispanic/Latino population has a higher adjusted risk of death than non‐Hispanic White people (Pinheiro et al. 2011). Additionally, Hispanic/Latino immigrants experience worsening health outcomes over generations, likely due to increased exposure to risk factors and the loss of protective factors despite socioeconomic gains (Gonzalez‐Guarda and Pearson 2024). Protective mechanisms such as strong family and social networks, cultural practices, ethnic pride, healthy coping skills, and belief in the “American dream” have been shown to buffer against acculturative stressors, including occupational stress (e.g., more work for less pay), family stress (e.g., intergenerational cultural gaps), and racial and ethnic discrimination (Gonzalez‐Guarda et al. 2023) and can potentially buffer against the stress associated with cancer diagnosis and treatment. More research is needed to elucidate protective factors across groups based on social identities and cultural resources.

We found higher levels of social support in the low symptoms group. High levels of social support are consistently linked to better physical and psychological health outcomes for patients with cancer (Golden and Earp 2012) and have been shown to positively influence quality of life, reduce symptom severity and cancer progression, enhance coping mechanisms and treatment adherence (Nausheen et al. 2009). Strong social support networks can mitigate cancer mortality and mobility, and psychological symptoms such as stress, depression, and anxiety, and also improve physical symptoms in patients with cancer, thereby aiding in more effective symptom management (Chambers et al. 2022; Mazzella et al. 2010). Psychosocial factors such as social support may affect levels of pain interference in patients with breast cancer (Fisher et al. 2021). Additionally, patients receiving palliative care for advanced cancer who have higher social support have been found to experience less pain intensity (Yang et al. 2024), which aligns with our study results highlighting the importance of social support in alleviating symptom occurrence among patients with CRC. Implementing social support strategies is essential for improving patient outcomes and promoting cancer care equity.

In addition to SDOH, access to symptom management medications may play an important but unmeasured role in shaping symptom severity. Patients’ ability to afford, obtain, and appropriately use supportive medications—such as antiemetics, antidiarrheals, analgesics, or appetite stimulants—can directly influence the severity and persistence of symptoms including nausea, vomiting, diarrhea, pain, and appetite loss. Prior work suggests that cost‐related medication underuse and nonadherence are common among patients with cancer and are associated with poorer symptom control and quality of life (Lu et al. 2021). Additionally, even when medications are accessible, inadequate health literacy or limited understanding of medication instructions can compromise their effective use (Halverson et al. 2015; Holden et al. 2021). These barriers, whether financial, educational, or system‐related, may disproportionately affect patients with fewer economic resources, limited health literacy, or inadequate access to supportive care services, potentially contributing to the disparities in symptom occurrence (Reshma et al. 2024).

Many studies have examined the relationship between diet quality and CRC incidence risk, but we believe that this is the first study to examine the relationship between diet quality and symptom occurrence. Healthy dietary patterns characterized by high intake of fruits, vegetables, whole grains, and lean meats or fish, along with low intake of red or processed meats and refined carbohydrates, have been associated with a reduced risk of CRC (Santarelli et al. 2008). For example, for both sexes combined, individuals in the highest quintile of the HEI score have demonstrated a significantly lower risk of colorectal, colon, and proximal colon cancers (Arthur et al. 2023). Among men, adherence to the HEI has been associated with a statistically significant reduction in risk for distal colon cancer, and women in the highest quintile of the HEI have demonstrated reduced risk for distal colon cancer (Reedy et al. 2008).

Although studies have examined the relationship between diet quality and cancer incidence, findings on symptom management are sparse. Cohort data from the Multiethnic Cohort Study, which identified 4770 invasive CRC cases, found that higher HEI scores were inversely associated with CRC risk across most racial and ethnic subgroups (Park et al. 2017). Added sugar has been identified as a high‐risk factor for CRC incidence and been associated with greater symptom occurrence, in some cases. Goncalves et al. (Goncalves et al. 2019) found that consumption of the equivalent of just one can of soda sweetened with high‐fructose corn syrup per day led to the development of more and larger tumors, without affecting weight; the authors concluded that high‐fructose corn syrup may promote colon tumor growth by fueling lipid synthesis within tumor cells, and through glucose absorption by blood vessels and fructose absorption directly in the colon. Additionally, the authors found that added sugar consumption may reduce gut microbial diversity, potentially influencing colon cancer development (Goncalves et al. 2019). This result suggests the importance of (a) limiting added sugar for the prevention and treatment of cancer and (b) considering the intersectionality of SDOH and diet quality to enhance understanding of CRC outcomes.

## 5 Limitations

This study has several notable limitations. First, the sample lacks diversity, with 80% of participants identifying as non‐ Hispanic White American, exceeding the national percentage of 65%–70% (National Cancer Institute 2026). Notably, our sample may underrepresent populations disproportionately affected by colorectal cancer, for example, Black patients (Siegel et al. 2026), which may limit the generalizability of our findings. The small representation of Hispanic individuals (10%) among ethnic groups requires careful interpretation, particularly as they comprise a small portion of the high symptom occurrence group. This limitation reflects the inherent nature of secondary analyses using electronic health record data, as the study sample consisted of insured. Consequently, the generalizability of the findings to more diverse or underserved populations may be limited. Nonetheless, this sample bias underscores the need to incorporate SDOH perspectives and to prioritize the inclusion of underrepresented populations in future research. Second, the participants predominantly had high levels of education, socioeconomic status, and health insurance coverage and these demographic factors and were limited in capturing the full context of SDOH social capital, as the data were derived from a hospital‐based dataset; this introduces potential bias in understanding the broader population, particularly in the context of SDOH. Future studies should aim to broaden the sample to include a more diverse population and comprehensive SDOH measures, potentially using national datasets. Third, the measures of diet quality used were designed for the general population and not specific to patients with CRC; therefore, these dietary components may not be ideal for patients with CRC who are undergoing surgery or in the process of receiving treatment. Developing CRC‐specific diet quality measures would enable the creation of tailored dietary interventions that address the unique needs of this population. Fourth, when diet quality–stratified participants were included in the analysis, the effective analytic sample was 137 of the 256 patients. This reflects an inherent limitation of using secondary analysis data; however, the associations we observed remained statistically significant and clinically meaningful. Future research using larger, prospectively collected datasets would strengthen the evidence base and further validate these findings. Fifth, we were unable to examine changes in diet quality over time because dietary data were collected at a single time point only. As with any dietary assessment, there is also a potential for recall bias, under‐ or over‐reporting, or challenges in accurately estimating portion sizes (Shim et al. 2014). Given the exploratory nature of this study, we did not apply corrections for multiple comparisons. While this approach allowed us to identify potential patterns and generate new hypotheses, it may also increase the likelihood of false‐positive results. Future studies with larger samples and confirmatory designs should consider multiple comparison adjustments to validate these findings. Lastly, this study was conducted within the U.S. healthcare context and is limited by a relatively small sample size, certain findings may still be informative for other settings. However, variations in healthcare systems, insurance structures, and availability of multidisciplinary supportive care may influence the extent to which these findings are transferable to countries outside of the United States.

This study provides strong evidence of the important role of social support and dietary composition in managing CRC symptoms during and after treatment. The symptom profiles identified in this study should be interpreted as exploratory and sample specific. Although these classes are not designed to function as standardized classification tools with predetermined thresholds, they effectively underscore significant variability in symptom experiences among CRC survivors. By linking symptom profiles to SDOH and diet quality, this study provides a conceptual framework for understanding how SDOH shape symptom experiences. Future research using larger and more diverse samples is needed to validate these profiles and examine their reproducibility across settings. Clinically, these results emphasize the necessity of tailored dietary interventions to improve symptom management and enhance quality of life for CRC patients. By integrating social and nutritional support into care plans, nurses and other healthcare providers can offer more comprehensive and effective symptom management strategies. This research has implications for developing tailored dietary interventions that not only address cancer incidence but also focus on managing symptoms through dietary strategies.

## 6 Conclusion

SDOH are intricately linked to health outcomes, yet structured SDOH factors such as access to healthcare services, insurance coverage, transportation, food security, and structural racism (National Academies of Sciences, E., & Medicine 2024) often present challenges that are not easily modifiable. Despite these complexities, nurses, and other clinicians, including physicians, dietitians, and pharmacists, can play a pivotal role in mitigating the impact of SDOH on patients' health. Strategies to address cancer inequities include implementing robust social support systems for patients and their families, delivering tailored dietary interventions for cancer patients, and promoting improvements in diet quality during and after treatment. Integrating these multidisciplinary, patient‐centered approaches into cancer survivorship care has the potential to improve symptom management, enhance quality of life, and advance health equity in patients with CRC.

## Author Contributions

Conceptualization: Youran Lee, Qing Yang, Melissa Kay, Donald E. Bailey, Rosa M. Gonzalez‐Guarda. Data Acquisition: Biljana Gigic, Cornelia M. Ulrich, Sheetal Hardikar, Adetunji T. Toriola, David Shibata, Christopher I. Li, Doratha A. Byrd, Jane C. Figueiredo. Ildiko Strehli, Megan Mclaws, Vaia Florou, Mmadili Ilozumba, Patricia Erickson, Apurva Medhe. Data Management: Youran Lee. Data Analysis: Youran Lee, Qing Yang. Writing – original draft preparation: Youran Lee. Writing – review and editing: Youran Lee, Rosa M. Gonzalez‐ Guarda, Qing Yang, Melissa Kay, Donald E. Bailey, Cornelia M. Ulrich, Biljana Gigic, Sheetal Hardikar, Doratha A. Byrd, Christopher I. Li,. Supervision: Rosa M. Gonzalez‐Guarda.

## Conflicts of Interest

The authors declare no conflicts of interest.

## Supporting information


**Table S1:** Flowchart of Sample Size at 6‐Month Time Point.

Supporting File

## Data Availability

The data that support the findings of this study are available on request from the corresponding author. The data are not publicly available due to privacy or ethical restrictions.
