# Supplementary material for: Distinct Symptom Occurrence Subgroups Among Patients With Colorectal Cancer: Differences in Social Determinants of Health and Diet Quality
Source: Res Nurs Health. 2026 May 26;49(5):595–611. doi: 10.1002/nur.70083 (PMC13539668; doi:10.1002/nur.70083)
Supplement: Supplementary file 1 [file NUR-49-595-s001.docx]

**Supplementary Table 1.** Flowchart of Sample Size at 6-Month Time Point

Having total HEI values

(N=876)

Having total social support values (N=1,788)

Having at least one symptom values (N=2,164)

Total number of dataset (N= 4,252)

Exclude:

Participants

who do not have all RUCA variables (n=3,332)

Exclude: Participants who do not have all demographic variables (n=0)

Exclude: Participants

who do not have any symptom variables

(n=3,169)

Exclude: Participants

who do not have any

social support (n=2,464)

Exclude: Participants

who do not have any

Health eating index (n=3,364)

Having at least one demographic values (N=4,252)

Having at least one RUCA (N=920)

Having at least one symptom values + demographics (N=1,083)

Having at least one symptom values + at least one demographics +all social support+ HEI_total_score (n=137)

Having at least one symptom values + at least one demographics + at least one RUCA (N=241)

Having at least one symptom values + at least one demographics +social support (n=256)

*Demographics/social support: age, gender, race, ethnicity, social support (n=231, no missing), education(n=200), income (n=200), marital status(n=205)

Having at least one symptom values + HEI_total_score+ all social support+ at least one demographic demographics+RUCA (N=35)

Having at least one symptom values +

all social support+ at least one demographics+RUCA (N=47)

***Demographics inclusion:** age, gender, education, income, race, ethnicity, marital status

*** RUCA variable inclusion**: urban/rural classification, primary RUCA code, secondary RUCA code (based on address & zip code), RUCA class source, population in the census tract, land area in the census tract, population density
