# Supplementary material for: Distinct Symptom Occurrence Subgroups Among Patients With Colorectal Cancer: Differences in Social Determinants of Health and Diet Quality
Source: Res Nurs Health. 2026 May 26;49(5):595–611. doi: 10.1002/nur.70083 (PMC13539668; doi:10.1002/nur.70083)
Supplement: Supplementary file 2 [file NUR-49-595-s002.docx]

**Appendix A.** Latent Class Analysis: Model fit Indices for the Specified Number of Classes

| **Model** | ***LogLik** | ***AIC** | ***BIC** | ***CAIC** | **Adjusted BIC** | **df** | **Entropy** |
| --- | --- | --- | --- | --- | --- | --- | --- |
| Class 2  C1 = 92  C2 = 164 | -1243.25 | 481.62 | 548.98 | 567.98 | 488.75 | 492 | 0.83 |
| Class 3  C1 = 49  C2 = 123  C3 = 84 | -1211.22 | 437.57 | 540.38 | 569.38 | 448.44 | 482 | 0.77 |
| Class 4  C1 = 82  C2 = 65  C3 = 61  C4 = 48 | -1192.55 | 420.23 | 558.49 | 597.49 | 434.85 | 472 | 0.77 |
| Class 5  C1 = 57  C2 = 27  C3 = 60  C4 = 46  C5 = 66 | -1181.91 | 418.93 | 592.65 | 641.65 | 437.31 | 462 | 0.89 |

*Abbreviations: AIC = Akaike’s Information Criterion; BIC = Bayesian Information Criterion; CAIC= Consistent Akaike Information Criterion; LL = log-likelihood
